# The Aqueous Leaf Extract of *M. oleifera* Inhibits PEDV Replication through Suppressing Oxidative Stress-Mediated Apoptosis

**DOI:** 10.3390/ani12040458

**Published:** 2022-02-13

**Authors:** Yanan Cao, Shuoshuo Zhang, Yanjie Huang, Shuai Zhang, Haifei Wang, Wenbin Bao

**Affiliations:** 1 Key Laboratory for Animal Genetics, Breeding, Reproduction and Molecular Design, College of Animal Science and Technology, Yangzhou University, Yangzhou 225009, China; yncao1994@163.com (Y.C.); zhangshuoshuo1108@163.com (S.Z.); m18252719706@163.com (Y.H.); shuai_zhang1990@163.com (S.Z.); hyfiwang@yzu.edu.cn (H.W.); 2 Joint International Research Laboratory of Agriculture and Agri-product Safety, Yangzhou University, Yangzhou 225009, China

**Keywords:** PEDV replication, aqueous leaf extract of *M. oleifera*, oxidative stress, apoptosis

## Abstract

**Simple Summary:**

The porcine epidemic diarrhea virus (PEDV), a porcine enteropathogenic coronavirus, can cause enormous economic losses in the swine industry. There is no effective commercial vaccine against PEDV infection. In this study, we found that an aqueous leaf extract of *M. oleifera* (MOE) exhibited antiviral activity in response to PEDV infection at the stage of PEDV replication instead of attachment or internalization. Mechanistically, MOE suppressed the oxidative stress and the expression of inflammatory cytokines induced by PEDV infection and upregulated the expression of anti-apoptotic proteins, which further led to less cell apoptosis. This study is the first report showing that MOE has antiviral potential as a new prophylactic and therapeutic strategy against PEDV infection.

**Abstract:**

Porcine epidemic diarrhea (PED), one of the serious enteric diseases caused by the porcine epidemic diarrhea virus (PEDV), is responsible for enormous economic losses in the global swine industry. However, available commercial vaccines fail to protect pigs from PEDV infection due to the appearance of PEDV variants. Hence, it is necessary to find an effective and cost-efficient natural product to protect pigs from PEDV infection. In this study, we first found that an aqueous leaf extract of *M. oleifera* (MOE) exhibited antiviral activity in response to PEDV infection. Furthermore, time-of-addition experiments revealed that MOE inhibited PEDV replication rather than attachment and internalization. Mechanistically, MOE significantly suppressed the production of reactive oxygen species (ROS) and malondialdehyde (MDA) induced by PEDV infection, and restored glutathione peroxidase (GSH-Px) activity. Importantly, the addition of MOE alleviated oxidative stress and the expression of inflammatory cytokines and resulted in fewer apoptotic cells during PEDV infection. These results indicated that MOE might be an effective anti-PEDV drug used to control PED disease and may be helpful in developing a new prophylactic and therapeutic strategy against PEDV.

## 1. Introduction

Porcine epidemic diarrhea (PED) is a devastating enteric disease caused by the porcine epidemic diarrhea virus (PEDV) infection in pigs, and the mortality can be up to 100% in newborn piglets, causing huge economic losses in the swine industry. PEDV usually infects small intestinal epithelial cells and causes pronounced villous atrophy, resulting in acute malabsorption syndrome with symptoms of watery diarrhea, vomiting, and anorexia. The interactions between PEDV and host factors have been well studied. Some cell surface proteins, including porcine aminopeptidase N (APN) [1], sialic acid [2], and a transferrin receptor (TFR1) [3], have been identified as the receptors to medicate PEDV invasion. Furthermore, host factors, including the early growth response gene 1 (EGR1) [4], IL-11 [5], and IFN-III [6], have been reported to have an anti-PEDV capability. However, these proteins are costly and difficult to apply to the swine industry. Despite several commercial PEDV vaccines being available, PEDV still persists around the world, including in China, and the appearance of variant PEDV strains makes the control of the virus become more difficult [7]. Therefore, it is necessary to explore and develop potential drug or feed additives to improve the immunity of pigs or prevent pigs from PEDV infection. 

PEDV contains four structural proteins, including spike (S), membrane (M), envolpoe (E), and nucleocapsid (N) proteins. The PEDV S protein can bind to the specific receptor and mediate the earlier stage of PEDV infection [8]. Additionally, the PEDV M protein is the most abundant structural protein and plays a crucial role in progeny PEDV assembly [9]. The PEDV E protein possesses the ability to inhibit the expression of type I interferon (IFN) during PEDV infection [10]. Furthermore, the N protein is the only protein present in the helical nucleocapsid and plays a crucial part in escaping host innate immunity by suppressing interferon-β (IFN-β) production and interferon-stimulating gene (ISG) expression [11]. PEDV infection consists of four phases: attachment, internalization, replication, and release. The first step of PEDV infection is mediated by the attachment of PEDV S protein to the specific host receptor. The next step of the PEDV life cycle is the translation of the PEDV replicase gene and the assembly of the viral replicase complexes. Following the replication and sub-genomic RNA synthesis, PEDV S, E, and M proteins are translated and further move into the endoplasmic reticulum-Golgi intermediate compartment (ERGIC). Finally, the viral genomes coated with N protein bud into membranes of the ERGIC, which contains the structural proteins, taking the shape of mature virions [12]. 

Medicinal plants are the biggest age-old source of therapeutically beneficial phytochemicals, which are not only used for preventing and treating many diseases of human beings, but also have the potential to be used as feed additives in livestock production [13,14]. Some natural products or their extracts have been reported to show anti-virus activities and improve the production performance of animals. The Moringa oleifera tree (*M. oleifera*) belongs to the family of Moringaceae and is originally from the north of India [15]. Due to its tolerance to various types of soils, *M. oleifera* is cultivated in different environments. Since *M. oleifera* is a remarkable species with high nutritional value and good biomass production, a previous study explored the utilization of *M. oleifera* as livestock fodder to improve animal production in goats, sheep, and cows. *M. oleifera* leaf meal improves the bowel health of broiler chickens owing to balancing intestinal microflora. *M. oleifera* leaf meal can replace a partial protein source in the poultry diet without causing any poor effects [16]. Furthermore, *M. oleifera* leaf meal is capable of improving pork quality and increasing the amount of unsaturated fatty acid in subcutaneous fat and meat, which contributes to improving human health. Additionally, the effects of *M. oleifera* leaf meal on pigs mainly depend on the growth stages [17]. Apart from being a feed additive, *M. oleifea* has been reported to possess an antiviral ability. Importantly, the crude extracts of *M. oleifera* decrease the level of HBV cccDNA in HepG cells [18], indicating that *M. oleifera* has the potential to inhibit viruses. Given that *M. oleifera* and its extraction are rich in nutrients and bioactive compounds, in this study we evaluated the anti-PEDV potential of an aqueous leaf extract of *M. oleifera* and explored the inhibitory mechanism on Vero cells, and attempted to provide evidence for application of *M. oleifera* leaves in the swine industry. 

## 2. Materials and Methods

### 2.1. Cell Lines, Virus, and Reagents

Vero E6 cells were bought from the National Center for Type Culture Collection, and PEDV CV777 strain was kindly provided by the China Agriculture University. Cells were cultured in DMEM medium supplemented with 10% fetal bovine serum (FBS, GIBCO, Australia) and 100 μg/mL penicillin/streptomycin in a humidified 5% CO_2_ incubator at 37 °C. PEDV stocks were generated in Vero E6 cells. In brief, Vero E6 cells were infected with PEDV at a multiplicity of infection (MOI) of 1 for 1 h at 37 °C, when cells had reached approximately 50–60% confluence. Then, the inoculum was removed and washed three times with phosphate-buffered saline (PBS). DMEM supplemented with 2% FBS was subsequently added, and incubation was continued at 37 °C for 72 h post-infection until the monolayer appeared to be completely involved with cytopathic effect (CPE). Then, the infected cells were subjected to three freeze–thaw cycles, and the suspension was clarified by centrifugation at 5000 rpm for 10 min. The supernatant was harvested and stored at −80 °C. The antibody against PEDV was purchased from Medgene labs (IF, 1:200; FACS, 1:100; Western-blot, 1:1000). The antibodies against Suivivin (A1551), B-cell lymphoma 2 (BCL2) (A2845), and Caspase-3 (A0214) were sourced from ABclonal (Wuhan, China). Rabbit anti-GAPDH (MB001), goat anti-rabbit IgG (H+L)-HRP (ZJ2020-R), and goat anti-mouse IgG (H+L)-HRP (ZJ2020-M) were from BioWorld Technology, Inc. (St. Louis Park, MN, USA). The aqueous leaf extract of *M. oleifera* was purchased from Undersun Biomedtech, Ltd. (Xi’an, China).

### 2.2. Cell Cytotoxicity Assay

The cytotoxic effect of aqueous leaf extract of *M. oleifera* was assessed by Cell Counting Kit-8 (CCK8) assay (C0038; Beyotime, Shanghai, China). Vero E6 cells were cultured at a density of 4 × 10^5^ cells/mL in 96-well plates in a humidified incubator (37 °C, 5% CO_2_) for 12 h and treated with different concentrations of the aqueous leaf extract of *M. oleifera* for 24 h. Then, the 10 μL/well CCK8 solution was added into each well of the plate for 2 h, and absorbance was measured at 450 nm on a Tecan Spark 10M microplate reader (Tecan, Zürich, Switzerland). The cell viability was determined as the ratio of the absorbance of the samples treated with different concentrations of the aqueous leaf extract of *M. oleifera* versus the untreated control. 

### 2.3. Quantitative Real-Time PCR

Cells were collected, and total RNA was extracted by RNAiso Plus (TaKaRa, Dalian, China), then reverse transcription was conducted using HiScript qRT SuperMix (R223-01; Vazyme, Nanjing, China). From three independent experiments, different gene expressions were calculated by using the comparative Ct method and normalized against *GAPDH* level. The primers used in this study were listed as follows (Table 1).

### 2.4. Western Blot Analysis

Cells were washed by ice-cold PBS, then lysed and harvested in Radio-Immunoprecipitation Assay buffer (RIPA buffer) with 1 mM phenylmethyl sulfonyl fluoride (PMSF). Protein concentrations were measured by BCA Kit (P0010S; Beyotime, Shanghai, China), and 20 μg samples were loaded in wells and separated by SDS-PAGE on 10% or 12.5% gels (Epizyme, Shanghai, China), and blotted into PVDF membranes (Millipore, MA, USA). Then, the blots were incubated with primary antibodies at 4 °C overnight and washed with Tris-buffered saline with Tween 20 (TBST). After extensive washing, the blots were incubated with HRP-labeled goat anti-rabbit or goat anti-mouse antibodies (Abs). Finally, the blots were exposed with a enhanced chemiluminescence (ECL) detection system (Vazyme, Najing, China).

### 2.5. Indirect Immunofluorescence Assay

To determine the antiviral activity of MOE on PEDV infection, Veo cells were fixed by using 4% paraformaldehyde for 10 min, 0.1% Triton X-100 in PBS was added for 5 min to improve the permeability of cell membrane, and 5% bovine serum albumin (BSA) was used to block the non-specific binding. After blocking, differently treated cells were incubated with PEDV primary antibodies (1:200) overnight at 4 °C. Cells were washed using PBS three times and then incubated with fluorochrome-conjugated secondary antibodies (1:200) for 30 min at room temperature; 1 μg/mL 4’, 6-diamidino-2-phenylindole (DAPI) was used to stain the nucleus for 5 min after washing. Images were captured using a Leica DMi8 fluorescence microscope (Leica, Wetzlar, Germany) and analyzed using Leica LAS AF Lite (Leica).

### 2.6. Flow Cytometry Assay

The cells (10^6^ cells) were harvested after pancreatin digestion and washed by PBS, and cells were fixed and permeabilized with a cellular fixation and permeabilization buffer set (Invitrogen, CA, USA). Then, cells were stained with the PEDV primary antibody (1:100) at room temperature for 30 min, then rinsed and incubated with fluorochrome-conjugated secondary antibodies (1:200), and then washed again three times with PBS. Finally, acquisition of the fluorescent cells was performed by BD FACSVerse^TM^ (Becton Dickinson, Franklin Lakes, NJ, USA), and the data were analyzed by FlowJo software (Version 10, Ashland, OR, USA). 

### 2.7. The Plaque-Forming Assay

Confluent monolayers of Vero cells inoculated the supernatant containing serial ten-fold dilutions of PEDV for 1 h in a humidified incubator (37 °C, 5% CO_2_), then the supernatant was removed and overlaid with 0.7% low-melting-point agarose in DMEM until the plaque formation at 37 °C. The plates were fixed with 4% formaldehyde, and the cells were stained with 1% crystal violet to visualize plaques. 

### 2.8. Measurement of Anti-Oxidative Stress Activity

Vero cells (10^6^ cells) were infected with PEDV in the presence of aqueous leaf extract of *M. oleifera* or equal volume of PBS for 48 h. To detect total cellular reactive oxidative species (ROS), a fluorescence indicator DCFH-DA (10 μM) (D6470; Solarbio, Beijing, China) was incubated with Vero cells at 37 °C for 20 min. After incubation, cells were washed three times in PBS and viewed under Leica DMi8 fluorescence microscope (Leica, Wetzlar, Germany). For GSH-Px and MDA measurements, the treated cells were lysed with cell lysis buffer for Western and IP (P0013; Beyotime, Shanghai, China) and measured by Cellular Glutathione Peroxidase Assay Kit (S0056; Beyotime, Shanghai, China) and Lipid Peroxidation MDA Assay Kit (S0131S; Beyotime, Shanghai, China) following the manufacturers’ instructions, respectively.

### 2.9. Cellular Apoptosis Assay

To evaluate the protective effect of aqueous leaf extract of *M. oleifera* on PEDV induced apoptosis, Vero cells were infected with PEDV in the presence of different concentrations of aqueous leaf extract of *M. oleifera* for 36 h. Equal PBS was treated as the mock control. The cellular apoptosis of different treated groups was measured by Annexin V-fluorescein isothiocyanate (FITC)/propidium iodide (PI) Apoptosis Detection Kit (A211-01; Vazyme, Nanjing, China). Briefly, the cells were harvested and resuspended in 100 μL binding buffer, then labeled with Annexin V and PI for 10 min. Finally, the stained cells were measured with flow cytometry (BD FACSVerse™, Becton Dickinson, Franklin Lakes, NJ, USA), and the data were analyzed using FlowJo software (Version 10, Ashland, OR, USA).

### 2.10. Statistical Analysis 

Statistical analysis was performed by Statistical Program for Social Sciences (SPSS 16.0, SPSS Inc., Chicago, IL, USA). Data are presented as the mean ± standard deviation (SD), and differences between control and experimental groups employed Student’s t-test and one-way analysis of variance (ANOVA). For all analysis, *p* values of <0.05 were considered statistically significant (* *p* < 0.05; ** *p* < 0.01; *** *p* < 0.001). 

## 3. Results

### 3.1. Cytotoxicity of the Aqueous Leaf Extract of M. oleifera on Vero Cells

**Figure 1:**
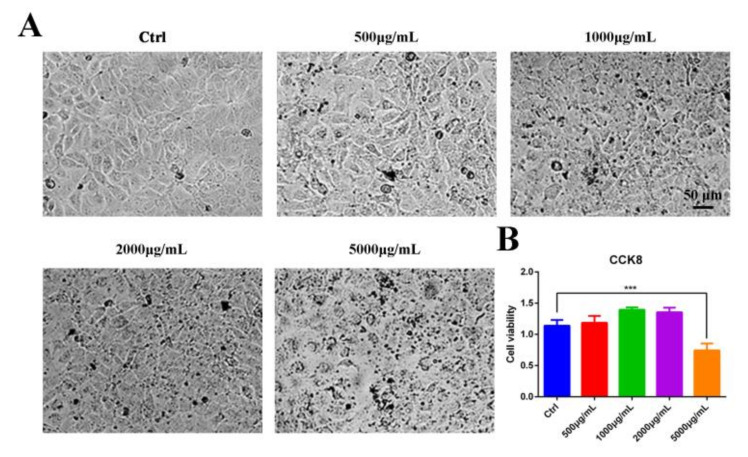
Cytotoxicity of MOE on Vero cells. (**A**) Light microscope of Vero cells treated with indicated concentrations of MOE at 37 °C for 24 h. (**B**) Vero cells were seeded in 96-well plates. After different concentrations of MOE ranging from 500 μg/mL to 5000 μg/mL were treated at 37 °C for 24 h and equal volume of PBS as Ctrl, cytotoxicity was determined by CCK8 assay. Data are expressed as three independent experiments. *** *p* < 0.001. MOE: the aqueous leaf extract of *M. oleifera*.

Vero cells, susceptible cells of PEDV, were used as a model *in vitro* to detect the safety of the aqueous leaf extract of *M. oleifera* (MOE). CCK8 assay was performed to determine the optional concentrations from 500 μg/mL to 5000 μg/mL of MOE. We found that the cytotoxic effect of MOE on Vero cells was dose-dependent, and MOE did not elicit any significant cytotoxic effect at concentrations of 500–2000 μg/mL (Figure 1A,B), indicating that MOE exhibited a high degree of safety.

### 3.2. Antiviral Activity of the Aqueous Leaf Extract of M. oleifera on Vero cells

**Figure 2:**
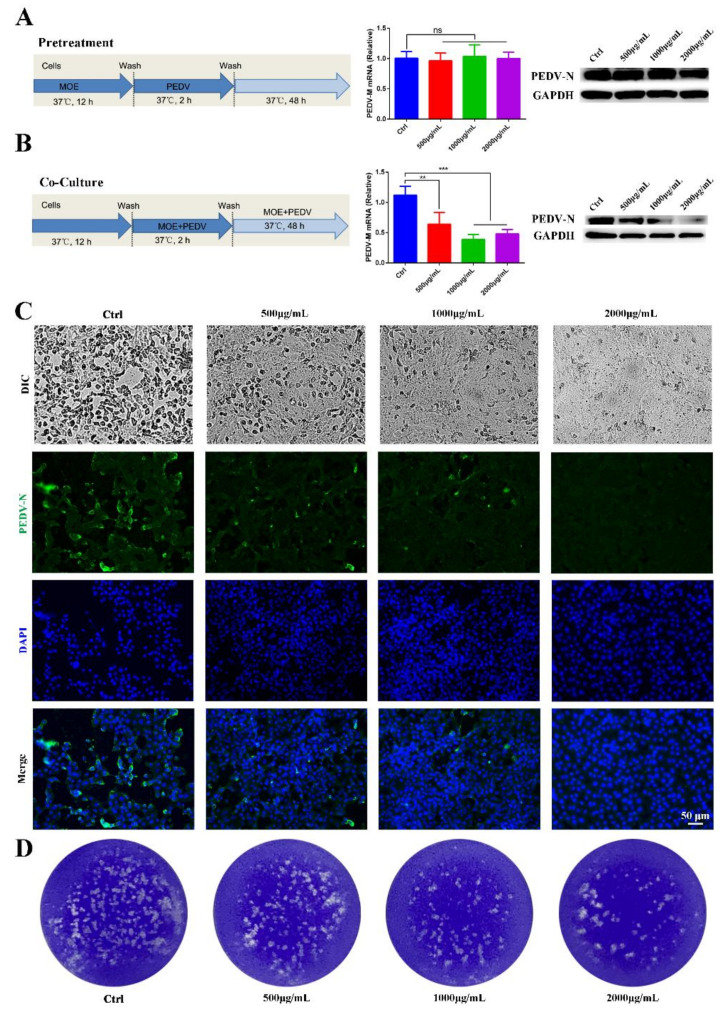
Antiviral activity of MOE on PEDV infection on Vero cells. Vero cells were pretreated with MOE at different concentrations of 500, 1000, and 2000 μg/mL or PBS for 12 h, respectively, then the cells were infected with PEDV (MOI = 0.1) for 2 h. After extensive washing, cells were cultured with or without MOE for 48 h. (**A**) Schematic of time-of-addition analysis of MOE (500, 1000, and 2000 μg/mL) treatment against PEDV infection on Vero cells using pretreatment models, PEDV-M mRNA was detected by qRT-PCR, and PEDV-N protein was detected by Western blotting. (**B**) Co-culture models. PEDV-M mRNA was detected by using qRT-PCR, and PEDV-N protein was detected by Western blotting. (**C**) The light microscope of Vero cell to observe cytopathic effect. Viral yields were titrated by IFA. Green: PEDV-N; blue: DAPI; scale bars, 50 μm. (**D**) The virions were detected by plaque assay. ** *p* < 0.01; *** *p* < 0.001; ns, no significance. MOI: multiplicity of infection; MOE: the aqueous leaf extract of *M. oleifera*; PEDV: porcine epidemic diarrhea virus.

To determine the appropriate dose range of MOE having anti-PEDV activity, Vero E6 cells were treated with 500, 1000, and 2000 μg/mL of MOE throughout the PEDV infection cycles, which included the pretreatment and during and after infection stages. Viral yields were assessed after 48 h using different experimental methods. Given that the PEDV M protein stands for the most abundant membrane protein, and during PEDV infection, the PEDV N protein presents the most abundant protein uniquely in the nucleocapsid, we chose these two proteins to explore the effect of MOE on PEDV infection. Firstly, pretreating Vero cells with MOE did not inhibit PEDV infection (Figure 2A). Subsequently, MOE was added during the PEDV infection stage to evaluate its antiviral effect. The PEDV-M mRNA level was significantly lower in the presence of MOE during PEDV infection (Figure 2B). Additionally, Western blotting also revealed that MOE decreased the expression of PEDV-N protein in a dose-dependent manner (Figure 2B), original whole Western blot figures see Supplementary File S1. In comparison with the PEDV infection group, the cytopathic effect (CPE) was lower in MOE-treated groups (Figure 2C), and the antiviral effect of MOE was further determined by an indirect immunofluorescence assay (IFA) (Figure 2C). Furthermore, a plaque assay was used to assess the alive virions released in the supernatant, and the number of the plaques was significantly less in the supernatants of cells treated with MOE than that with PEDV treated alone (Figure 2D). These results illustrated that treatment of Vero cells with 500, 1000, and 2000 μg/mL MOE concentrations led to a gradual decrease in PEDV infection.

### 3.3. Aqueous Leaf Extract of M. oleifera Impaired PEDV Replication Instead of Attachment or Internalization

**Figure 3:**
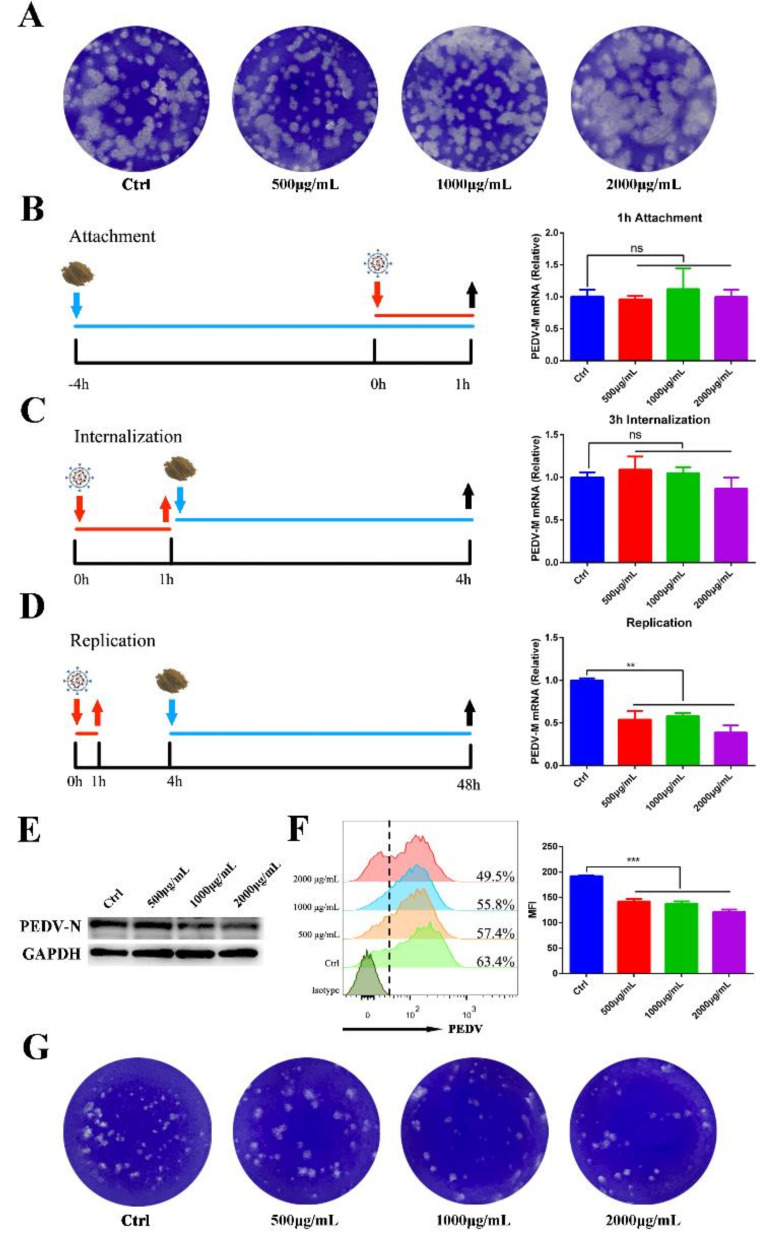
Effect of MOE on the inactivation, attachment, entry, and replication of PEDV. (**A**) Inactivated assay. PEDV was incubated with indicated concentrations of MOE at 37 °C for 1 h; then, the mixture was added into confluent monolayers of Vero cells to detect the alive virions numbers by plaque assay. (**B**) Virus attachment assay. The supernatant was replaced with MOE during virus attachment stage. PEDV-M mRNA level was detected by qRT-PCR. (**C**) Virus internalization assay. The supernatant was replaced with MOE during virus internalization stage. (**D**) Virus replication assay. The supernatant was replaced with MOE during virus-replication stage. PEDV-M mRNA level was detected by qRT-PCR. (**E**) Cell lysates were subjected to Western blotting assay. (**F**) Cells were subjected to flow cytometry. (**G**) Virus in the supernatant was detected by plaque assay. Data are expressed as three independent experiments. ** *p* < 0.01; *** *p* < 0.001; ns, no significance. MOE: the aqueous leaf extract of *M. oleifera*; PEDV: porcine epidemic diarrhea virus; MFI: mean fluorescence intensity.

To further explore the mechanism of MOE on affecting PEDV infection, we first tested whether different concentrations of the extraction (500, 1000, and 2000 μg/mL) are directly virucidal and inactive PEDV particles. The plaque assay was performed after co-incubation of MOE and PEDV, and the result showed that MOE treatment failed to affect the viral activities (Figure 3A). Next, a time-of-addition assay was performed to detect which stage of MOE blocks PEDV infection. Vero cells were treated with MOE or an equal volume of PBS at 37 °C for 4 h, and then exposed to PEDV particles at 4 °C for 1 h. After extensive washing, the treated cells were collected to extract RNA to assess the effect of MOE on PEDV attachment through qRT-PCR. No significant difference in the PEDV-M mRNA level was observed, indicating that MOE did not affect PEDV attachment to Vero cell (Figure 3B).

In evaluating the effect of MOE on PEDV internalization, Vero cells were infected with PEDV for 1 h at 4 °C, then the supernatant was replaced with different concentrations of MOE or an equal volume of PBS, and cells were cultured at 37 °C for 3 h. As shown in Figure 3C, MOE did not impede PEDV internalization. Since that MOE did not affect the attachment and internalization of PEDV to Vero cells, we suspected that MOE might impair PEDV replication. Given this hypothesis, MOE was added to the supernatant during the PEDV replication stage. The levels of PEDV-M mRNA and PEDV-N protein were significantly reduced in MOE-treated groups in a dose-dependent manner (Figure 3D,E). Flow cytometry also further confirmed that MOE hindered PEDV replication (Figure 3F); the percentage of PEDV positive cells and the mean fluorescence intensity (MFI) was significantly lower in MOE-treated groups than that in the PEDV-infection group. Besides, the virion numbers were significantly less in indicated concentrations of extraction than in the PEDV-infection group (Figure 3G). Taken together, these results implied that MOE primarily inhibited PEDV infection through inhibiting virus replication instead of attachment or internalization.

### 3.4. Aqueous Leaf Extract of M. oleifera Impaired PEDV Replication by Inhibiting Apoptosis

**Figure 4:**
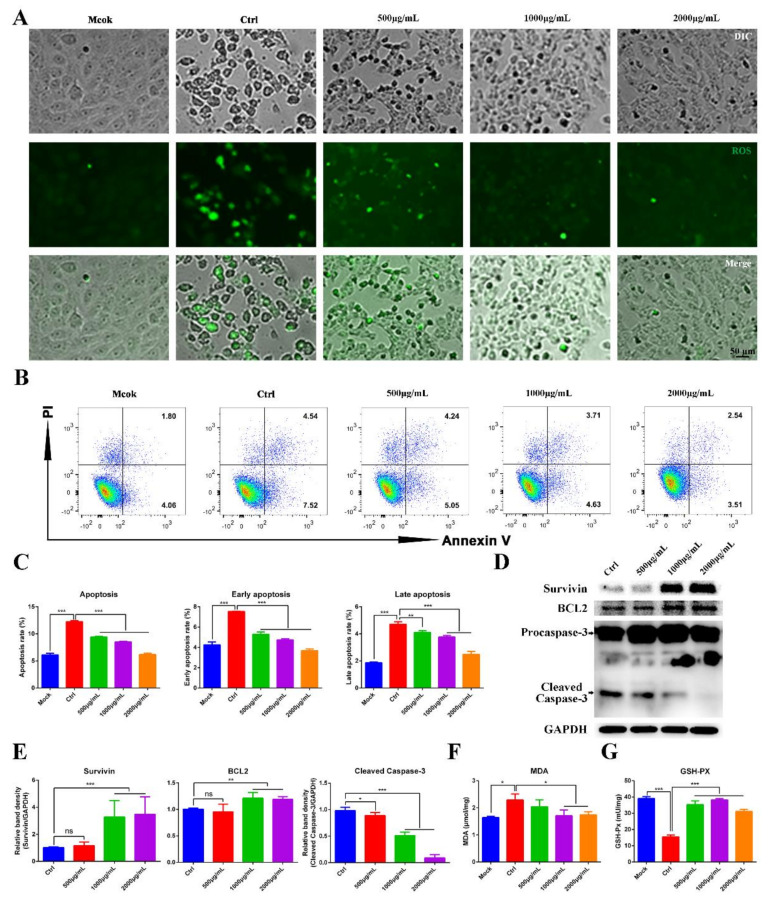
MOE inhibited PEDV replication by suppressing PEDV induced apoptosis. (**A**) Vero cells were infected with PEDV (MOI = 0.1); after extensively washing, the medium was replaced with different concentrations of MOE for 48 h. Then, the cellular ROS was detected by DCFH-DA (10 μM, 20 min). (**B**) Apoptosis was quantified by combined staining with Annexin V and PI, and the fluorescence was analyzed at 36 h by flow cytometry. (**C**) Total apoptosis rate, early apoptosis, and late apoptosis rate were detected. Early apoptosis cells (Annexin V-positive); late apoptosis cell (positive for Annexin V and PI). Early apoptosis plus late apoptosis equals total apoptosis. (**D**) Survivin, BCL2, and Caspase-3 were detected by Western blotting. (**E**) The intensity of the bands (Survivin, BCL2, and cleaved Caspase-3) in terms of density was measured and normalized against GAPDH expression. (**F**) MDA production. (**G**) GSH-Px activity. Data are expressed as three independent experiments. * *p* < 0.05; ** *p* < 0.01; *** *p* < 0.001; ns, no significance. MOE: the aqueous leaf extract of *M. oleifera*; PEDV: porcine epidemic diarrhea virus; ROS: reactive oxygen species.

PEDV infection can induce autophagy and apoptosis in Vero cells by a reactive-oxygen-species (ROS)-dependent signaling pathway [19,20]. Therefore, we wondered whether MOE inhibits PEDV replication by inhibiting ROS production. ROS production was evaluated by using the fluorescent detection probe DCFH-DA. ROS was observed during PEDV infection, while MOE decreased the ROS production in PEDV-infected Vero cells, indicating MOE may impair PEDV replication through the ROS-dependent signaling pathway (Figure 4A). Additionally, ROS induces lipid peroxidation and further promotes apoptosis. To further evaluate the effect of MOE on lipid peroxidation, malondialdehyde (MDA), the product of lipid peroxidation, was detected by an MDA assay kit. As shown in Figure 4F, PEDV infection significantly increased the production of MDA, and MOE downregulated the production of MDA. Moreover, the content of total glutathione peroxidase (GSH-Px), which eliminates ROS and the products of lipid peroxidation, was assessed; the results showed that MOE restored the GSH-Px activity, which was inhibited by PEDV infection (Figure 4G).

We sought to determine whether MOE has the potential to inhibit Vero cell apoptosis induced by PEDV infection through flow cytometry. Upon PEDV infection, Vero cells underwent an early or late stage of apoptosis at 36 h, as indicated by the increase in the percentage of Annexin V-positive or AnnexinV- and PI-positive cells. As expected, MOE supplementation effectively reduced cell apoptosis (Figure 4B,C). Caspase-3 plays a critical role in the execution phases of the apoptosis of cells [21]. Hence, the apoptosis-related proteins, the cleaved Caspase-3, Survivin, and B-cell lymphoma 2 (BCL2) were detected by Western blotting. As Figure 4D,E showed, the treatment of Vero cells with MOE significantly counteracted the proapoptotic effect induced by PEDV infection and upregulated Survivin and BCL2. These results revealed that MOE inhibited PEDV infection by suppressing oxidative stress and further inhibited apoptosis induced by PEDV. 

### 3.5. Aqueous Leaf Extract of M. oleifera Alleviated the Inflammatory Cytokines Induced by PEDV Infection

**Figure 5:**
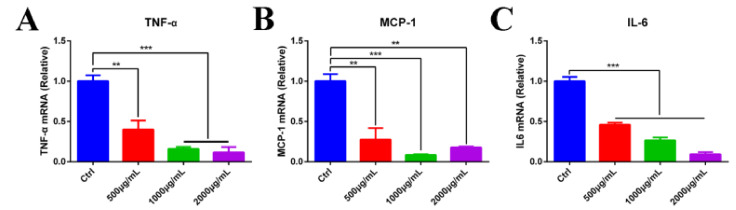
MOE inhibited the expression of inflammatory cytokines induced by PEDV infection. Vero cells were infected with PEDV (MOI = 0.1); after extensive washing, the cells with MOE in the supernatant were incubated at 37 °C for 48 h. (**A**) *TNF-α*, (**B**) *MCP-1*, and (**C**) *IL-6* mRNA levels were evaluated by qRT-PCR. Data are expressed as three independent experiments. ** *p* < 0.01; *** *p* < 0.001. MOI: multiplicity of infection; MOE: the aqueous leaf extract of *M. oleifera*; PEDV: porcine epidemic diarrhea virus.

Inflammatory cytokines were upregulated in response to PEDV infection. To further explore the mechanisms of the antiviral capability of MOE, expression levels of inflammatory cytokines were evaluated by qRT-PCR. MOE downregulated PEDV induced the high mRNA level of *TNF-α, IL-6,* and *MCP-1* (Figure 5A–C), indicating that MOE inhibited the inflammatory response stimulated by PEDV.

## 4. Discussion

PED is a disease that causes significant economic damage to the global swine industry and is one of the most devastating porcine viruses [22]. PEDV causes acute diarrhea, dehydration, and high mortality in neonatal piglets. Compared with older pigs, newborn piglets are more susceptible to PEDV. To date, there are three main PEDV strains in the world. CV777 strain, isolated in 1978 [23], belongs to the classic G1-genotype strain, and since late 2010, the emergence of a highly virulent G2-genotype resulted in enormous economic losses in the swine industry. Insertion-deletion (INDEL)-strains of PEDV have also been found [24]. Despite many efforts, the appearance of variant strains makes prevention and treatment of PEDV more difficult. Therefore, exploring a convenient and cost-effective method to protect pigs from PEDV infection is important to the pig industry. In this study, we found the aqueous extraction of *M. oleifera* leaves has the ability to inhibit PEDV infection. Moreover, we also proved that the *M. oleifera* suppressed PEDV replication rather than attachment and internalization. Importantly, *M. oleifera* inhibited the production of ROS and MDA induced by PEDV infection and alleviated oxidative stress by increasing the activity of GSH-Px. Overall, the aqueous leaf extract of *M. oleifera* suppressed the oxidative stress induced by PEDV infection.

Currently, traditional medicines play a significant part in treating virus infection [25,26]. Intriguingly, they can exert their effects in different stages of virus infection. Honeysuckle is believed to have an anti-virus effect, and a honeysuckle-encoded atypical microRNA2911 directly targets Influenza A Viruses with a broad spectrum, and the synthetic microRNA2911 could target H1N1-encoded PB1 and NS1 expression [27]. Additionally, an aqueous honeysuckle extract suppresses dengue virus replication and pathogenesis [28]. Tomatidine, a steroidal alkaloid from tomatoes, inhibits PEDV replication by inhibiting the activity of 3CL protease [29]. Some herbs and plants, due to their cost-effective and convenient features, have a wide prospect as animal feed additives in pig industries. *M. oleifera*, as a growth promoter, has been used as a natural feed supplement in the poultry and rabbit industries. Leaves of *M. oleifera* are rich in nutrition; studies have found that supplementary *M. oleifera* leaves improved the production performance of pigs, including increasing average daily gain, decreasing feed to gain ratio, and backfat thickness. Additionally, recent studies demonstrated that *M. oleifera* showed antimicrobial activity [30] and anti-HBV infection [31]. In this study, we first found that an aqueous leaf extract of *M. oleifera* (MOE) exhibited anti-PEDV ability in vitro. The plaque assay indicated that MOE failed to be virucidal. As for PEDV, the S protein, a glycoprotein assembled into homotrimers, medicates the attachment and membrane fusion through its S1 and S2 subunits [32]. However, supplementation with MOE did not influence the attachment and internalization of PEDV to Vero cells, which may indicate that MOE does not affect the expression of host receptors or the functions of host receptors and PEDV S protein. Moreover, we found that MOE impaired PEDV infection during the replication phase, indicating that MOE might block the transcription of the PEDV N gene. Overall, we indicated that MOE suppressed PEDV infection, primarily inhibiting PEDV replication.

The *M. oleifera* leaf and its aqueous leaf extract are rich in bioactive compounds. It is reported that MOE is rich in quercetin and its derivatives, and rutin [33,34]. Quercetin is widely distributed in vegetables and fruits [35], and rutin is the essential component in many traditional medicines such as Shuanghuanglian and Lianhuaqingwen [36]. Importantly, quercetin has the potential to inhibit influenza A virus (IAV) infection through interacting with the HA2 subunit of IAV, and evidence also showed that the rutin could impede IAV replication [37,38]. Moreover, quercetin has the ability to block Ebola virus infection through counteracting VP24 function [39]. Moreover, a molecular docking study indicated that quercetin might inhibit PEDV replication through binding to the active site and pocket of PEDV 3C-like protease [40]. Rutin can interact with the main protease (Mpro) of SARS-CoV-2 to inhibit virus infection [36]. Hence, based on the antiviral effect of these two compounds, we assumed that quercetin and rutin existed in MOE might have the anti-PEDV activity. 

ROS takes part in ER stress and is involved in numerous signaling pathways [41,42]. It is known that ROS is usually a toxic product during virus infection [43]. It is reported that PEDV infection can induce ROS production and further lead to ER stress [20]. Thus, we assumed that MOE suppresses PEDV infection by maintaining redox homeostasis. Given this hypothesis, related indicators of oxidative stress and apoptosis-related proteins were detected. We found that PEDV induces the production of ROS in Vero cells, while supplementation with MOE downregulated ROS production. Moreover, the MDA assay, which detects lipid peroxidation, also demonstrated that PEDV infection effectively perturbed redox homeostasis, while MOE alleviated MDA accumulation induced by PEDV. Furthermore, studies reported that the whole PEDV and PEDV S1 protein contributes to Vero-E6 cells’ apoptosis [44]. As a consequence of ROS and MDA accumulation, apoptosis usually occurs. Our study confirms that PEDV infection induced the production of ROS and, in turn, led to lipid peroxidation; furthermore, cell apoptosis occurred due to the accumulation of the products of oxidative stress. Notably, we found that MOE reduced the production of ROS and MDA. Moreover, MOE reversed the phenomenon of the PEDV-induced increase in the apoptosis rate and the decrease in the anti-apoptotic proteins, Survivin and BCL2 [45,46]. Our study suggests that MOE might inhibit PEDV replication by regulating oxidative stress and by upregulating anti-apoptotic levels.

## 5. Conclusions

In brief, our results demonstrate that the aqueous leaf extract of *M. oleifera* has the capability to inhibit PEDV infection during the PEDV replication stage in vitro. Furthermore, *M. oleifera* suppressed PEDV infection by suppressing the oxidative stress and apoptosis induced by PEDV infection. This work provides evidence that the aqueous leaf extract of *M. oleifera* may be useful as a feed additive in inhibiting PEDV infection, and further studies in vivo are needed to confirm it.

## Figures and Tables

**Table 1:** Primers sequences for qRT-PCR.

Primers	Sequences	Product Length
*IL-6*	F: AACCAACCACAAATGCCAG	77 bp
	R:GAGATGCGTCGTCATGTCCT	
*TNF-α*	F:GAAAGCATGATCCGGGACG	158 bp
	R:ATCACTCCAAAGTGCAGCAGA	
*MCP-1*	F:GCTTAATGGCACCCCATCCT	84 bp
	R:GAAGCAGTGGGTCAGGACAA	
*GAPDH*	F:ACATCATCCCTGCTTCTACTGG	188 bp
	R:CTCGGACGCCTGCTTCAC	
*PEDV-M*	F:AGGTCTGCATTCCAGTGCTT	216 bp
	R:GGACATAGAAAGCCCAACCA	

## Data Availability

The datasets generated during the current study are available from the corresponding author upon reasonable request.

## Supplementary Materials

The following supporting information can be downloaded at: https://www.mdpi.com/article/10.3390/ani12040458/s1, Supplementary File S1: Original whole Western blot analysis.
