# Supplementary figures and images for: The Aqueous Leaf Extract of M. oleifera Inhibits PEDV Replication through Suppressing Oxidative Stress-Mediated Apoptosis
Source: Animals (Basel). 2022 Feb 13;12(4):458. doi: 10.3390/ani12040458 (PMC8868277; doi:10.3390/ani12040458)

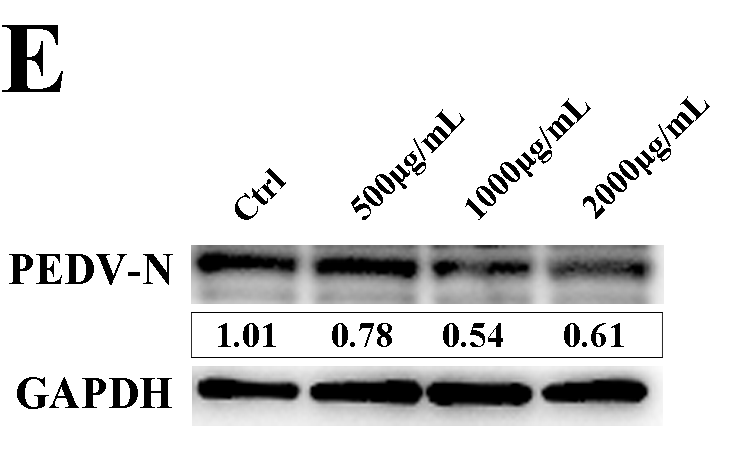

Supplement: Supplementary file 1 [file animals-12-00458-s001.zip › animals-1481486/Figure (3E).tif]

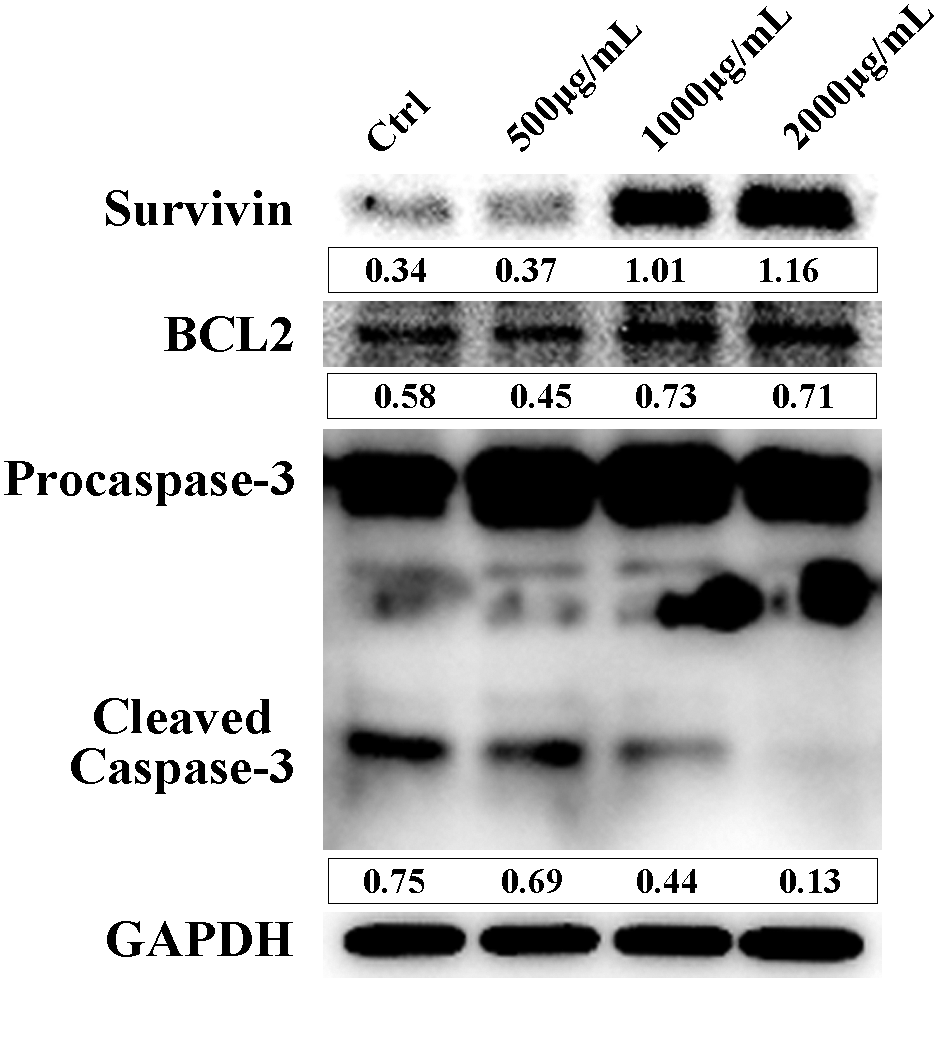

Supplement: Supplementary file 1 [file animals-12-00458-s001.zip › animals-1481486/Figure (4D).tif]

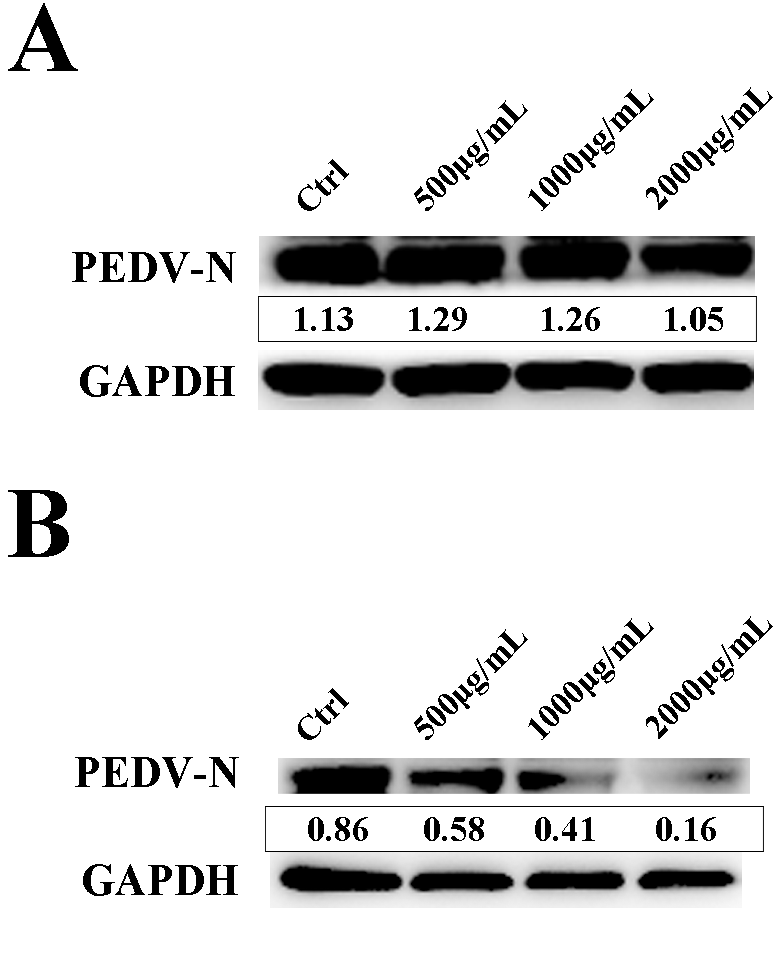

Supplement: Supplementary file 1 [file animals-12-00458-s001.zip › animals-1481486/Figures (2A and 2B).tif]

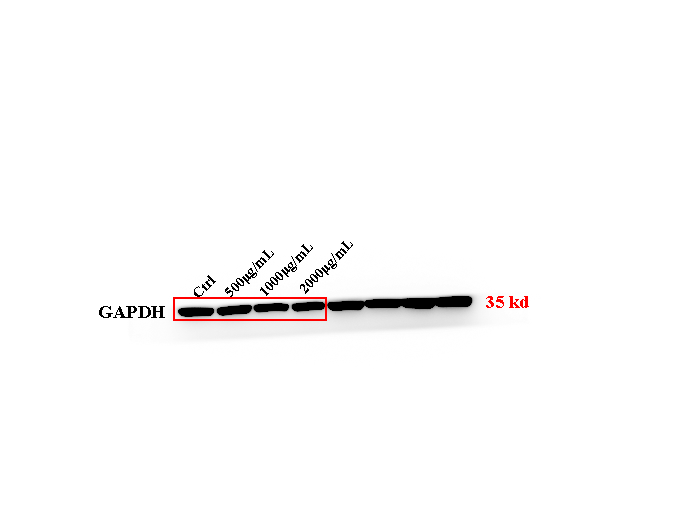

Supplement: Supplementary file 1 [file animals-12-00458-s001.zip › animals-1481486/WB Figures (2A)/Figure 2A GAPDH 2.tif]

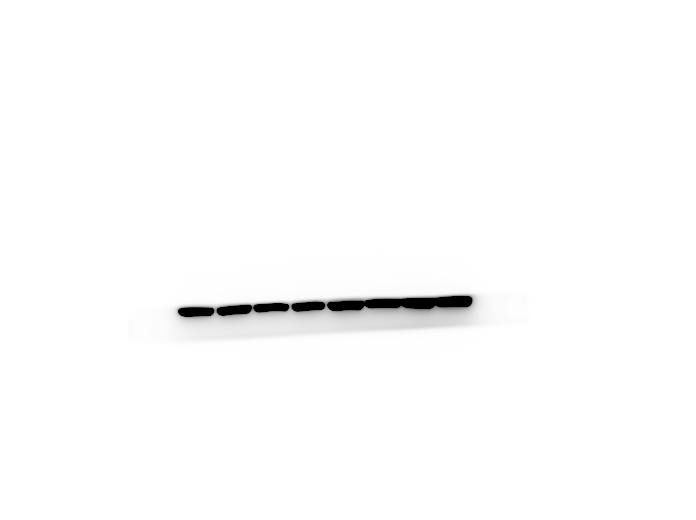

Supplement: Supplementary file 1 [file animals-12-00458-s001.zip › animals-1481486/WB Figures (2A)/Figure 2A GAPDH.tif]

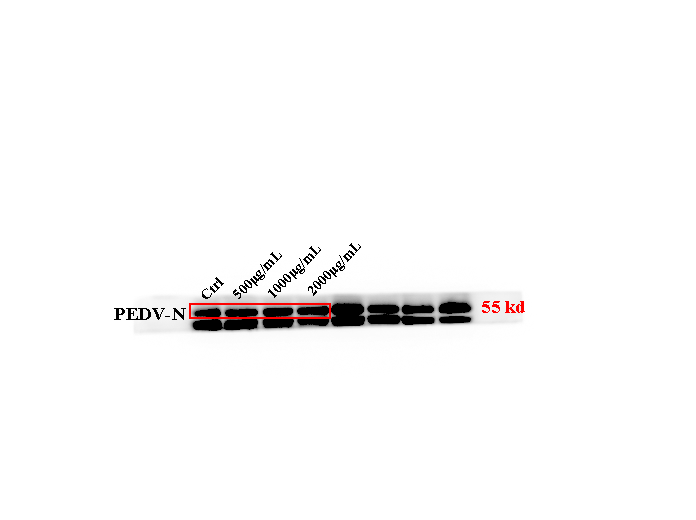

Supplement: Supplementary file 1 [file animals-12-00458-s001.zip › animals-1481486/WB Figures (2A)/Figure 2A PEDV-N 2.tif]

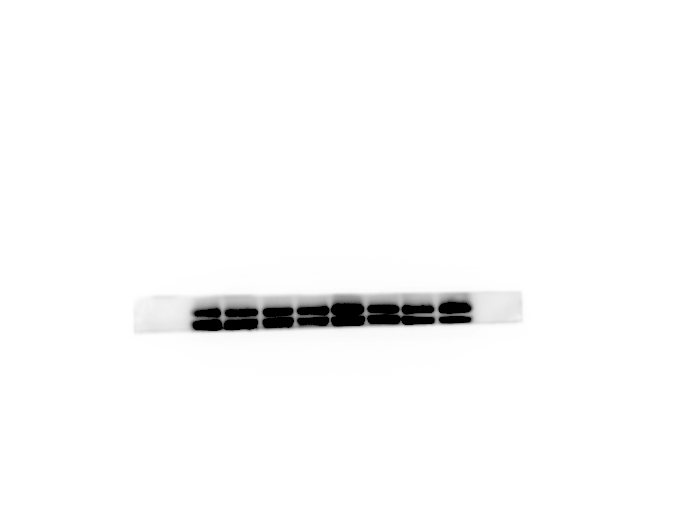

Supplement: Supplementary file 1 [file animals-12-00458-s001.zip › animals-1481486/WB Figures (2A)/Figure 2A PEDV-N.tif]

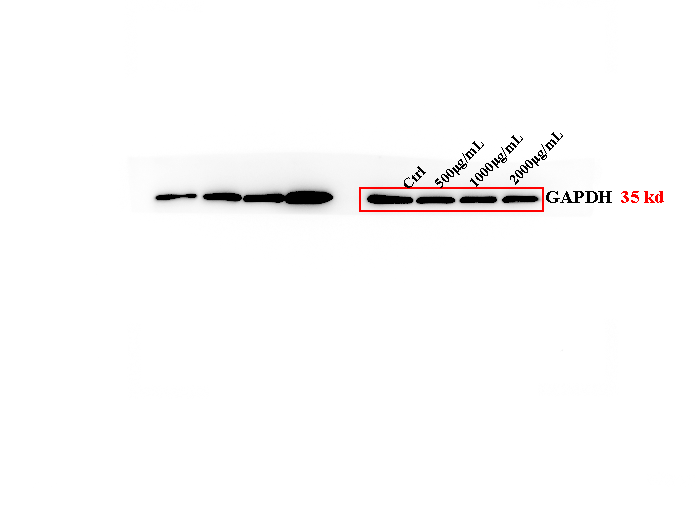

Supplement: Supplementary file 1 [file animals-12-00458-s001.zip › animals-1481486/WB Figures (2B)/Figure 2B GAPDH 2.tif]

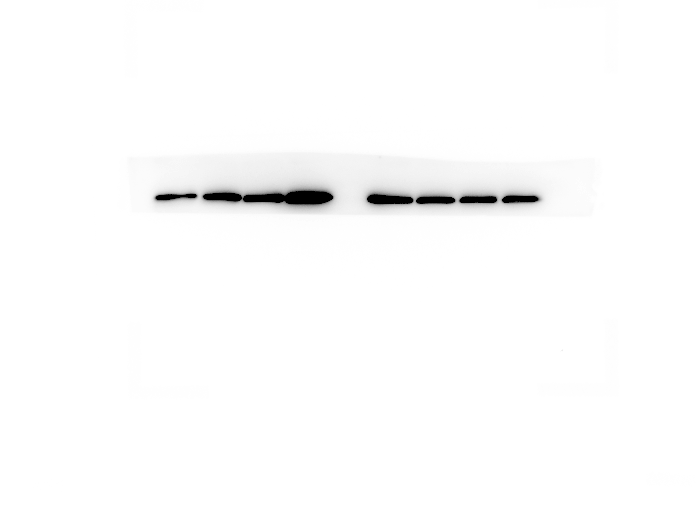

Supplement: Supplementary file 1 [file animals-12-00458-s001.zip › animals-1481486/WB Figures (2B)/Figure 2B GAPDH.tif]

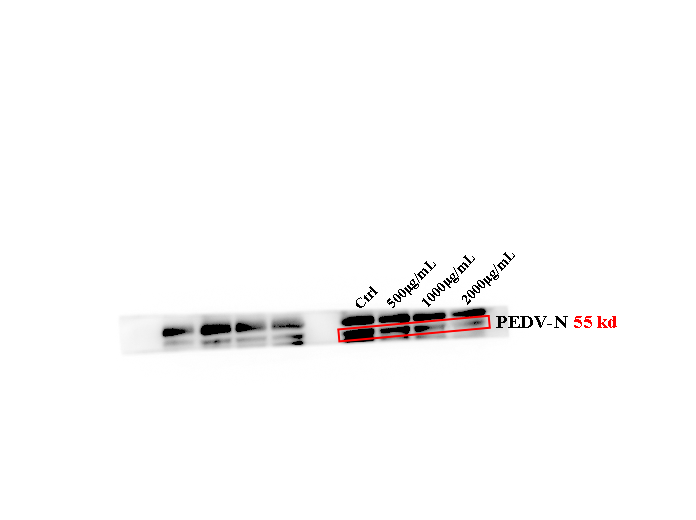

Supplement: Supplementary file 1 [file animals-12-00458-s001.zip › animals-1481486/WB Figures (2B)/Figure 2B PEDV-N 2.tif]

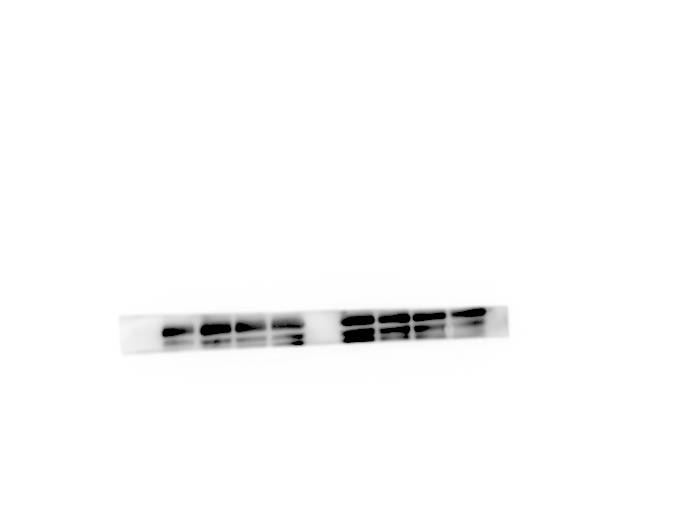

Supplement: Supplementary file 1 [file animals-12-00458-s001.zip › animals-1481486/WB Figures (2B)/Figure 2B PEDV-N.tif]

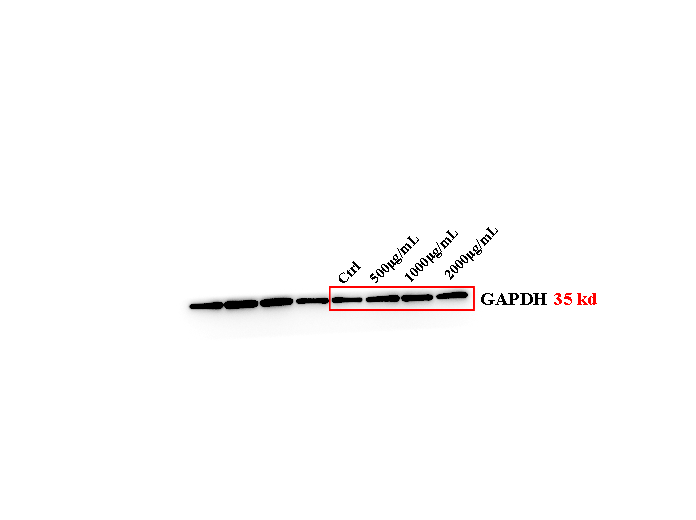

Supplement: Supplementary file 1 [file animals-12-00458-s001.zip › animals-1481486/WB Figures (3E)/Figure 3E GAPDH 2.tif]

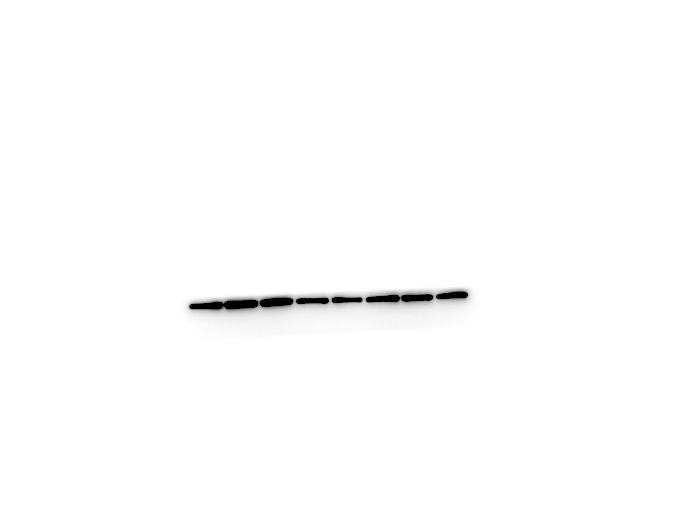

Supplement: Supplementary file 1 [file animals-12-00458-s001.zip › animals-1481486/WB Figures (3E)/Figure 3E GAPDH.tif]

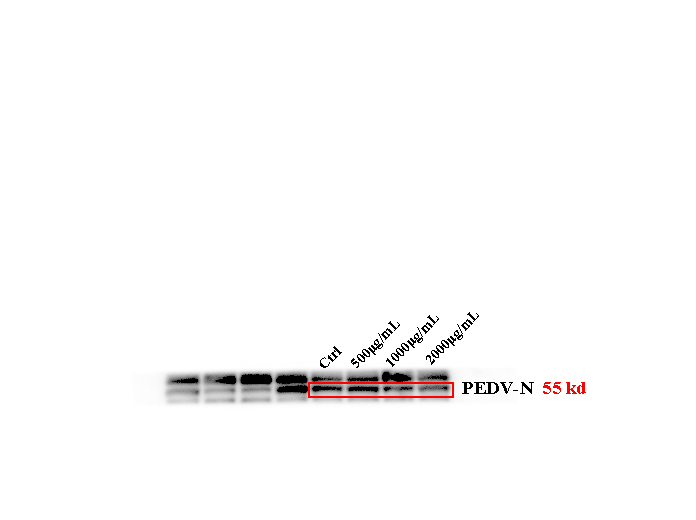

Supplement: Supplementary file 1 [file animals-12-00458-s001.zip › animals-1481486/WB Figures (3E)/Figure 3E PEDV-N 2.tif]

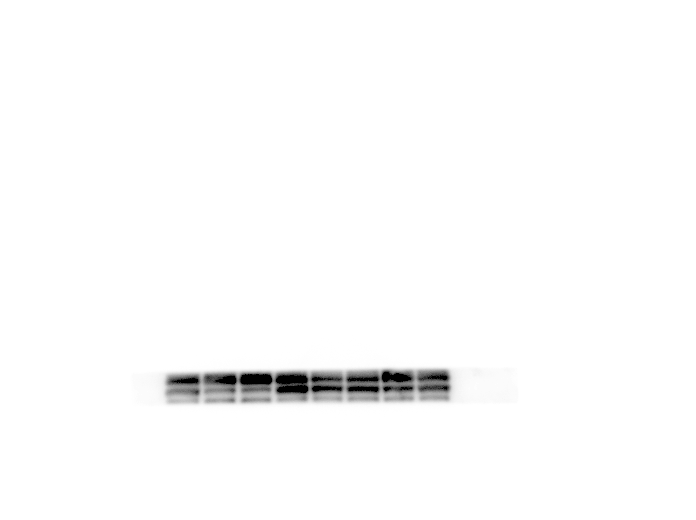

Supplement: Supplementary file 1 [file animals-12-00458-s001.zip › animals-1481486/WB Figures (3E)/Figure 3E PEDV-N.tif]

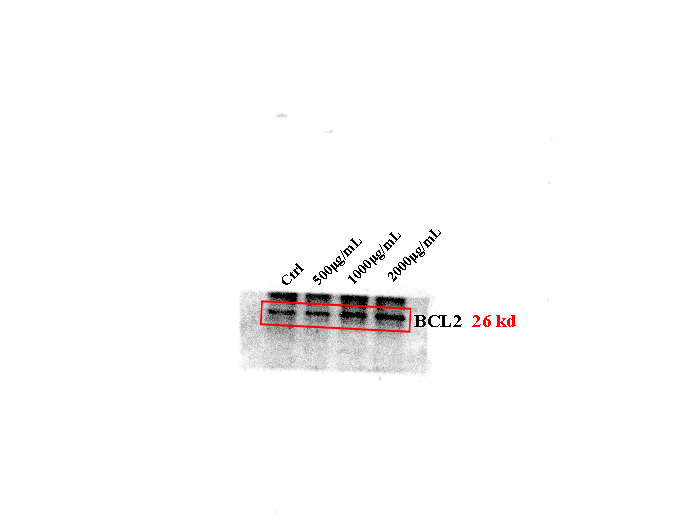

Supplement: Supplementary file 1 [file animals-12-00458-s001.zip › animals-1481486/WB Figures (4D)/Figure 4D BCL2 2.tif]

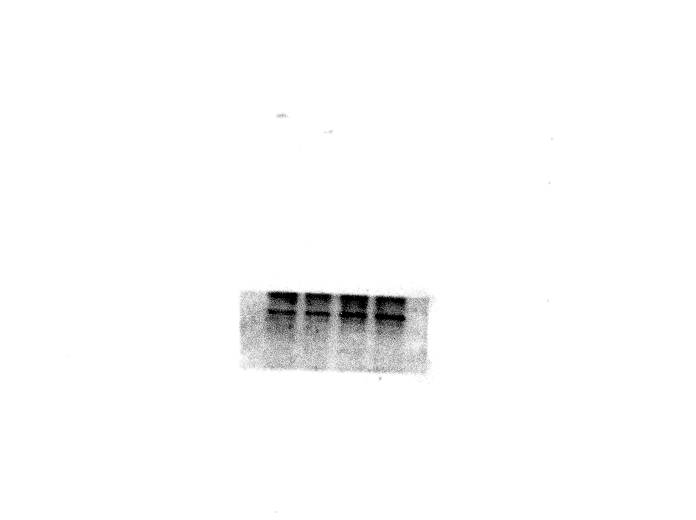

Supplement: Supplementary file 1 [file animals-12-00458-s001.zip › animals-1481486/WB Figures (4D)/Figure 4D BCL2.tif]

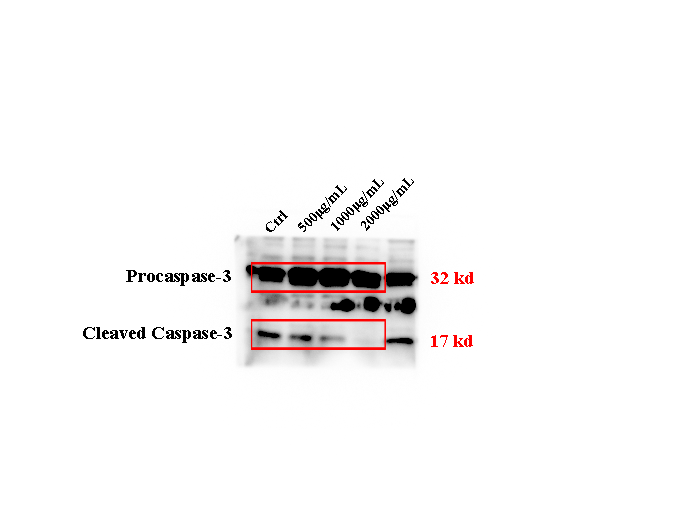

Supplement: Supplementary file 1 [file animals-12-00458-s001.zip › animals-1481486/WB Figures (4D)/Figure 4D Caspase3 2.tif]

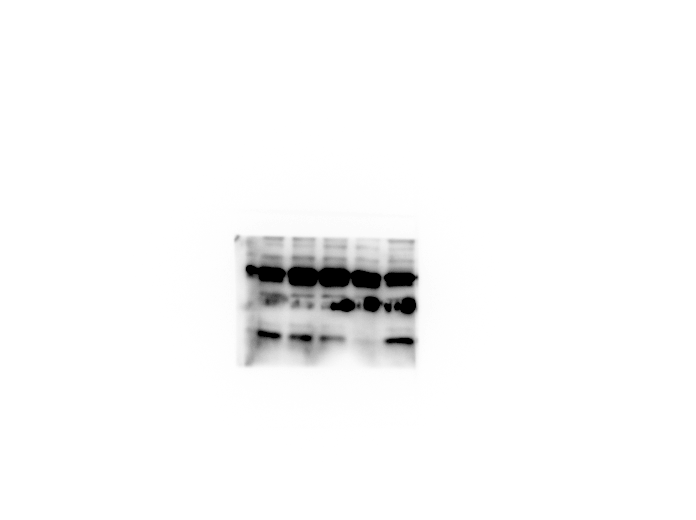

Supplement: Supplementary file 1 [file animals-12-00458-s001.zip › animals-1481486/WB Figures (4D)/Figure 4D Caspase3.tif]

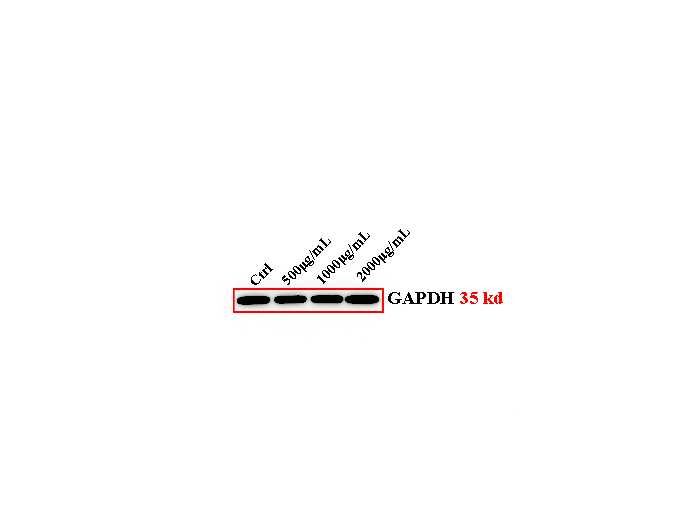

Supplement: Supplementary file 1 [file animals-12-00458-s001.zip › animals-1481486/WB Figures (4D)/Figure 4D GAPDH 2.tif]

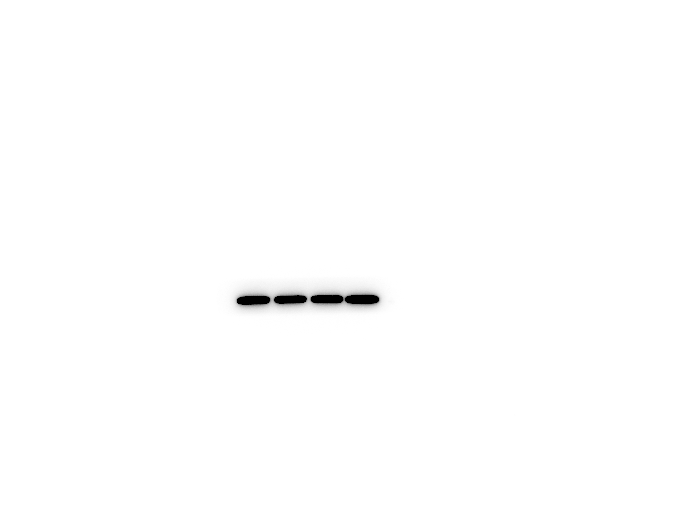

Supplement: Supplementary file 1 [file animals-12-00458-s001.zip › animals-1481486/WB Figures (4D)/Figure 4D GAPDH.tif]

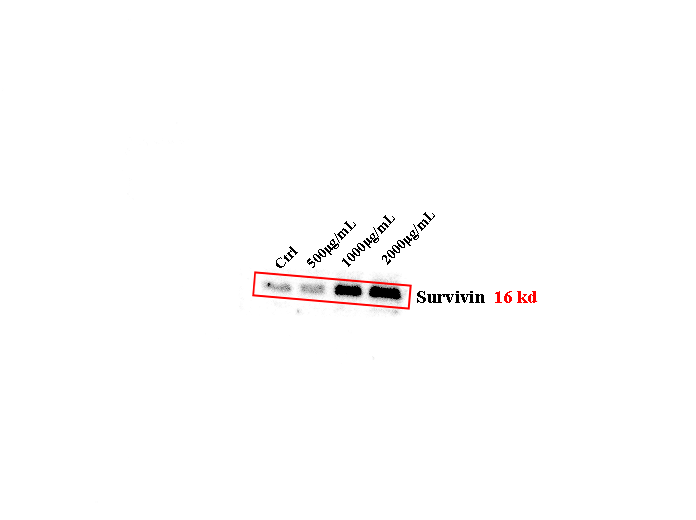

Supplement: Supplementary file 1 [file animals-12-00458-s001.zip › animals-1481486/WB Figures (4D)/Figure 4D Survivin 2.tif]

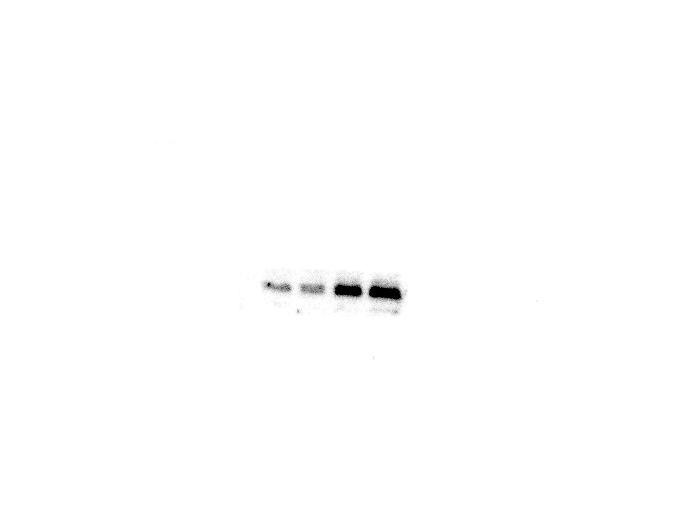

Supplement: Supplementary file 1 [file animals-12-00458-s001.zip › animals-1481486/WB Figures (4D)/Figure 4D Survivin.tif]
